# Lactoferrin for COVID-19 prevention, treatment, and recovery

**DOI:** 10.3389/fnut.2022.992733

**Published:** 2022-11-07

**Authors:** Ecem Bolat, Furkan Eker, Merve Kaplan, Hatice Duman, Ayşenur Arslan, Sümeyye Saritaş, Arif Sercan Şahutoğlu, Sercan Karav

**Affiliations:** ^1^Department of Molecular Biology and Genetics, Çanakkale Onsekiz Mart University, Çanakkale, Turkey; ^2^Department of Chemistry, Çanakkale Onsekiz Mart University, Çanakkale, Turkey

**Keywords:** lactoferrin, COVID-19, SARS-CoV-2, inflammation, iron homeostasis, spike proteins, ACE2 receptor

## Abstract

Severe Acute Respiratory Syndrome Coronavirus-2 (SARS-CoV-2), a unique beta-coronavirus, has caused the most serious outbreak of the last century at the global level. SARS-CoV-2 infections were firstly reported in the city of Wuhan in China in 2019 and this new disease was named COVID-19 by World Health Organization (WHO). As this novel disease can easily be transmitted from one individual to another *via* respiratory droplets, many nations around the world have taken several precautions regarding the reduction in social activities and quarantine for the limitation of the COVID-19 transmission. SARS-CoV-2 is known to cause complications that may include pneumonia, acute respiratory distress syndrome (ARDS), multi-organ failure, septic shock, and death. To prevent and treat COVID-19, some significant studies have been conducted since the outbreak. One of the most noticeable therapeutic approaches is related to a multifunctional protein, lactoferrin. Lactoferrin (Lf) is an 80 kDa cationic glycoprotein that has a great range of benefits from improving the immunity to antiviral effects due to its unique characteristics such as the iron-binding ability. This review summarizes the characteristics of SARS-CoV-2 and the potential applications of Lf for the prevention, treatment, and recovery of COVID-19.

## Introduction

### Severe Acute Respiratory Syndrome Coronavirus-2 (SARS-CoV-2) and COVID-19

Viruses, causing seasonal epidemics and sometimes pandemic outbreaks, have circulated between humans and animals throughout history. In the past centuries, various viruses adversely affected human health and evenly caused death. The Spanish flu in 1918, for instance, killed 50 million people around the world. In addition, swine flu in 2009 caused the death of around 4–5 million people. Nowadays, we have been waging war with a pandemic that is arising from the coronavirus family (1). In December 2019, Severe Acute Respiratory Syndrome Coronavirus-2 (SARS-CoV-2) infections reported as a cluster of pneumonia cases in city Wuhan in China. The newly discovered disease was named COVID-19 by World Health Organization (WHO) in 2020 (2).

Coronaviruses come from a large family known as the Coronaviridae. The name corona is derived from the Latin word “corona” meaning “crown” and was given to the virus due to having a crown-like appearance on its membrane called a spike (3). Human and animal coronaviruses have been describe in the literature before the COVID-19 outbreak (4). The virus is known to be genetically similar to previous viruses that emerged in China as SARS-CoV (79%) (in 2002) and in Saudi Arabia as MERS-CoV (50%)—Middle East Respiratory Syndrome Coronavirus (in 2012) (5, 6).

Coronaviruses are divided into four genera: α, β, δ, γ. While both alpha-type and beta-type coronaviruses infect mammals, gamma-type coronaviruses infect avian species, and delta-type coronaviruses are known to infect both type of species (7). SARS-CoV, MERS-CoV, and SARS-CoV-2 were result of the beta-type coronavirus activity eventually caused global pandemics (5).

Shortly after the emergence of COVID-19 in China, causing a global health crisis on a scale that has not been evidence in the last century (8). In the first days of January 2020, the reported number of patients that were diagnosed with COVID-19 infection was reported to be only 41 (9). Just within a few weeks, on January 30th, 2020, WHO declared the outbreak of COVID-19 as a Public Health Emergency of International Concern (PHEIC) (10). On July 8th, 2020, the coronavirus spread across 216 cities worldwide. Globally, as of 28th June 2022, there have been 542,188,789 confirmed cases of COVID-19 including 6,239,275 deaths, reported to WHO.

According to an epidemiological update published by WHO in February 2022, globally there are 422 million cases followed with 5.8 million deaths (11). The current data shows even higher numbers with the updated version in near end of the August 2022, the COVID-19 pandemic that has extend to Europe (246,729,836), Americas (174,625,662), Western Pacific (81,762,210), South-East Asia (59,908,896), Eastern Mediterranean (22,946,608), and Africa (8,777,310), has still causing serious numbers of deaths (12). In England, between October 2020 and April 2021, people that got COVID-19 positive were classified according to their conditions; Individuals with conditions such as hypertension (15%), diabetes mellitus (8.6%), chronic respiratory disease (21.2%) at greater risk to exposed with coronavirus (12, 13).

#### The genetic and physical structure of SARS-CoV-2

Since December 2019, many studies have been carried out to better understand SARS-CoV-2 genomic background. Several scientists tried to identify the source of this virus to analyze its genes and family tree. The fact that the virus has a unique RNA makes it possible to identify the virus and at the same time the properties of the SARS-CoV-2 could be come out thanks to this genome information. It was discovered that coronaviruses contain a single positive-stranded RNA as genetic material (5). They can jump from animals to humans (called a “spillover”) due to their high tendency of mutation (5). Therefore, it can be said that coronaviruses are “zoonotic” viruses (14). Furthermore, the genome sequence of SARS-CoV-2 is very similar to the type of coronavirus found in bats, which brings the idea that this virus's ancestors are bat viruses that eventually end up with its encountering with human species.

Coronaviruses' genome consist of the largest amount, 26.4–31.7 kb, between the RNA viruses (6). The genome is designed into six or seven regions, and each region contains at least one open reading frame (ORFs). These regions are separated by the presence of some sequences that have a signal(s) for transcription of multiple subgenomic mRNAs (7). SARS-CoV-2 has 6 ORFs and can encode 4 structural proteins which are nucleocapsid (N) protein, membrane (M) protein, spike (S) protein, and envelope (E) glycoprotein together with 16 non-structural proteins (1, 15, 16). Nucleocapsid, membrane and spike proteins are encoded by the peerless regions of the mRNAs (7). Among these structural proteins, the spike protein illustrates large protrusions from the virus surface, creating the crown appearance of coronaviruses with its two subunits, S1 and S2 (17). Apart from mediating virus entry into the host cell, spike protein is a crucial determinant of viral host spacing and tissue tropism, and the major stimulator of host immune responses (18). The spike proteins situate on the virus's membrane and bind to angiotensin-converting enzyme 2 (ACE2) which was found to be a receptor for the SARS-CoV-2 that is utilized by a virus-infected host cell, allowing the viral entry of the virus through endocytosis (16, 19). The ACE2's binding site is found in the S1 part of the spike protein, in the receptor binding domain (RBD) (17). ACE2 is mainly present in the lung, heart, kidney, liver, intestine, and other tissues (16, 19, 20). ACE2 does not only act as a virus receptor it also regulates and controls blood pressure, the functions of the heart and kidney (19).

#### The major effects of COVID-19 on human health

COVID-19 can adversely affect human health by infecting the respiratory tract. The most common symptoms observed are headache, smell and taste dysfunctions, dizziness, and impaired consciousness (21). Also, that is stated, these symptoms are not specific to SARS-CoV-2 infection. On the contrary, they are similar to symptoms observed in many other viral infections. SARS-CoV-2 can primarily be transmitted from person to person through respiratory droplets directly or by contacting contaminated surfaces that include droplets of someone who is infected with the virus (1). An infected person can release these droplets by coughing, talking, sneezing, or breathing (22). On contrary, others experience acute respiratory distress syndrome (ARDS) which the lungs cannot provide the body with enough oxygen because of injury to the alveoli in the lungs (23, 24). At the same time, the symptoms are appeared to be more severe in people with additional risk factors such as old age, obesity, diabetes, high blood pressure, heart disease, cancer, and chronic respiratory diseases (8). Moreover, COVID-19 patients were analyzed by their neurological symptoms with their frequency (25). The study showed that SARS-CoV-2 was detected in the cerebrospinal fluid due to the passage of the virus through the blood-brain barrier.

#### Diagnostic and therapeutic studies against COVID-19

Several types of tests are used to diagnose this infection such as reverse transcriptase-polymerase chain reaction (PCR) recognizes the virus based on its genetic fingerprint. An antibody test can also be applied to check the presence of antibodies produced by the host's immune system to identify foreign molecules such as viral spike proteins (26). Several types of vaccines have been asserted during the COVID-19 pandemic, such as messenger RNA-based, DNA-based, protein-based, viral-vectored, live-attenuated, and inactivated vaccines (27). Each type has some advantages and disadvantages in its production or action way.

For instance, nucleic acid vaccines are genetically considered a safer approach, thus, it provides a non-infectious approach for stimulating a stronger immune response compared to other traditional vaccines (28). Multiple parameters go around during the vaccination process such as continual production of safe and effective vaccines or being able to supply and deploy these vaccines worldwide (29). Viruses have a tendency to mutate frequently, which results in different variants (30) that disturb vaccine production. Therefore, vaccine's effectiveness might loss its influence against new variants. In this perspective, the development of mRNA vaccines that focus on spike proteins could be considered a more stabilized approach. However, the potential mutation on the spike protein puts these vaccines in the danger zone as well. Therefore, whenever a new persistent variant spreads through the population, each vaccine must be specifically put into test to confirm its efficiency has remained against the new variant.

For instance, after its detection in India, the delta variant started a huge concern amongst authorities due to its 60% higher transmissibility (31). Recently, the cases in the United States were dominated by the delta variant, followed by the cases in The United Kingdom, so on (32–35). Similarly, the alpha variant has been reported to have a 56% increased spreading rate (36). Even though it is stated that this variant has low or no effect on the vaccine's effectiveness, the increase in their spreading rate still causes some problems.

As a result, new alternatives or supportive approaches for diagnosis and treatment are always consider in high demand. Several distinct methods were applied to find alternative approaches to control the COVID-19 pandemic throughout its course. For instance, the spreading of COVID-19 was simulated with mathematical modeling for creating a decent foresight about the duration of the pandemic (31). Thus, it is of the utmost importance to consult some other potential methods and integrate them for assisting our current case in the pandemic.

### Lactoferrin—a multifunctional glycoprotein

Lactoferrin (Lf), which also called lactotransferrin, is a cationic glycoprotein with a molecular weight of about 80 kDa (37, 38). Lf is a multifunctional protein that has numerous biological functions include its antiviral and antibacterial effects as well as immunity booster tendencies owing to its characteristics (39, 40). Lf is a part of the transferrin family and has a 60% sequence identity with serum transferrin protein. The protein has a high binding affinity (K_d_ ~ 10^−20^ M) for Fe^+3^ ions (37, 41). In addition, it has three different isoforms; these are lactoferrin-α, lactoferrin-β, and lactoferrin-γ. While lactoferrin-α acts as an iron-binding agent but has no ribonuclease activity, both lactoferrin-β and lactoferrin-γ have ribonuclease activities but are unable to bind iron ions (38, 42). Lf includes 692 amino acids and composed of two α-helixes that connect globular lobes (37, 38, 42). Each N-lobe and C-lobe have a metal ion binding site where the metal ions, such as Cu^2+^, Zn^2+^, Mn^3+^, Al^3+^, and most importantly, Fe^3+^ can bind (43).

Lf bears noticeable importance due to several characteristic properties (Table 1), especially iron-binding ability, among other members of the transferrin family (66). Lf has a high resistance to releasing the iron ions at low pH values. As a result, the iron ions binding capability and keeping them inside the infected tissues with low pH is secured (42).

**Table 1:** Biological effects of lactoferrin.

**Effect type**	**Form of lactoferrin**	**Action mechanism**	**References**
Antiviral	Intact and/or peptides	Direct interaction with virus surface, DNA, or cell surfaces	(44–55)
Antibacterial	Intact and/or peptides	Iron binding, direct interaction with surface of bacteria	(44, 52–57)
Antifungal	Intact and/or peptides	Iron binding, direct interaction with surface of fungi	(53, 55, 58)
Enhancing immunity	Intact	Enhancement of natural killer cell activity and T-cell responses	(53, 55, 59–61)
Anti-inflammatory	Intact and/or peptides	Suppressing extracellular traps from neutrophils, polarization of macrophages to M2 type, inhibition angiotensin II pro-inflammatory activity	(62–64)
Iron homeostasis	Intact and/or large fragments	Iron binding, restoring levels of iron-binding proteins	(53, 55, 65)
Antiparasitic	Intact and/or peptides	Reducing the infectivity of parasites	(54)

Thus, the protein has a crucial role in infected and inflamed areas where it binds the iron ions, inhibits bacterial growth (bacteriostatic), and reduces their proliferation by taking the iron used in the bacterial growth from the environmental matrixes (67–69). This ability of Lf also known as its anti-microbial activity affects the growth and proliferation of a sort of infectious microorganisms from viruses to fungi (38, 67). During the antiviral activity, Lf also acts as an obstacle that binds to the viral cell surfaces with either receptors or co-receptors. Consequently, an act of viral attachment to the cell's surface is prevented (70), which enables Lf to have several positive influences on the immune system and the ability to fighting with viral infections (42).

### The therapeutic potential of lactoferrin to COVID-19

A variety of Lf effects in microbial and viral infections led to a suggestion that the iron-binding affinity of Lf gives the protein critical importance and role in inflammatory processes. The iron balance between the blood and tissues has a crucial significance, besides, Lf can create or protect this iron balance for patients that are infected by COVID-19 (41). At the same time, Lf has an immunomodulatory role and plays critical role by stimulating the cells involved in innate and acquired immunity, while increasing human and animal immunity against viral and bacterial diseases (71). Furthermore, Lf is known to have effects on plasminogen which is a system that is essential for the degradation of fibrin clots, activation of growth factors, removal of protein aggregates, and cell migration. Lf has anti-thrombin activity by binding directly to human plasminogen activation that may occur on the cell surface. As a result, some virulent bacterial species cannot bind to the human plasminogen and not penetrate the host cell membrane due to the competition with human lactoferrin (hLf) (72). Thus, hLf is known to reduce the frequent coagulation problem in patients exposed to COVID-19 (73).

Iron is also crucial for oxygen transport and helps in most biological functions by acting as an electron acceptor and donor for energy production (74). The optimum iron balance between tissues and blood is referred to as iron homeostasis, in which Lf possesses an important role on it (41). Iron homeostasis of Lf disrupted in the situation of a viral infection and inflammation. As a result, the intercellular iron concentration has increased cause a positive effect on viral replication (75). The chelation ability of Lf can be used to decrease the disease severity. The ability of stimulation, and remodeling iron proteins, can be used to reduce pro-inflammatory cytokine levels (75).

Bovine lactoferrin (bLf) is known to have the same functions and sequence homogeneity as hLf that is found in human milk and secretions (76, 77). It has confirmed that bLf can stop the infection in the early phase by decreasing serum ferritin, D-dimers, and IL-6 levels (73).

Due to those specialties of Lf, several clinical trials and treatment applications were developed for COVID-19 disease during the last couple of years (78, 79).

In Tables 1, 2, the type of viruses and effects of Lf against them were mentioned. During the search for treatment agents against COVID-19, these studies might point to the potential usage of Lf as a treatment agent due to its wide range of effects. Against SARS-CoV-2, there are lack of evidence and study that demonstrates these effects on the table will be also appear in COVID-19 treatment. The Lf's mechanisms that observed in other viruses involves a wide-spectrum, which is making Lf valuable choice of study. The studies that were performed with these backing informations are still limited and under investigation.

**Table 2:** Studies of different effects of lactoferrin on different viruses.

**Effect of lactoferrin**	**Target virus**	**Study type**	**References**
Inhibition of viral entry	Herpes simplex virus 1 and 2 (HSV-1 and HSV-2)	*In-vitro*	(55, 80)
	SARS-CoV		
Reducing severity and duration of infection	SARS-CoV-2	Clinical	(81)
Inhibition of cytopathic effect	Adenovirus	*In-vitro*	(82, 83)
Antiviral activity	Avian flu—H5N1	*In-vitro*	(82–86)
	Human papillomavirus (HPV)		
	Hepatitis B virus (HBV)		
	Mayarovirus (MAYV)		
Inhibition of binding and replication	Echovirus 5	*In-vitro*	(18, 46, 82, 83, 87)
	Hepatitis B virus (HBV)		
	Japanese encephalitis virus (JEV)		
	Mouse norovirus (MNV)		
Inhibition of viral replication	Cytomegalovirus	*In-vitro*	(83, 88)
	Echovirus type 6		
Inhibition of viral adsorption and increase survival	Enterovirus 71—EV71 Bovine viral diarrhea virus (BVDV)	*In-vitro, in-vivo*, clinical	(82, 83, 89–91)
	Herpes simplex virus type-1 (HSV-1)		
	Epstein-Barr virus (EBV)		
Neutralizing virus, blocking invasion	Hepatitis C virus	*In-vitro* and clinical	(83, 92, 93)
Inhibition of cytopathic effect	Human papillomavirus	*In-vitro*	(82, 94)
Inhibition of cytotoxicity, reduction in gastroenteritis incidence and symptom	Norovirus	*In-vitro* and clinical	(82)
Inhibition of cytopathic effect, decreasing the prevalence and severity	Rotavirus	*In-vitro* and clinical	(82, 95)
Blocking viral entry and inhibition of replication	Human immunodeficiency virus (HIV)	*In-vitro*	(83, 96, 97)
	Human parainfluenza virus type 2 (hPIV-2)		

A better understanding of the effect of Lf on the immune system is crucial for developing certain treatments, especially during the pandemic. A variety of Lf has been observed in recent studies (4, 98). With the knowledge of the known effects of Lf, it is possible to acknowledge the beneficial effects of the Lf treatment on COVID-19 patients. Still, the possibility of these effects is under examination. For the movement of wider treatment steps, a variety of studies and evidence are still required for Lf's influence on COVID-19 treatment. Particular research and investigations on Lf might lead to changing the patterns of possibilities into realities, so the accurate explanation of the potential mechanisms lie underneath.

#### Antiviral mechanism and potential clinical uses of lactoferrin against COVID-19

**Figure 1:**
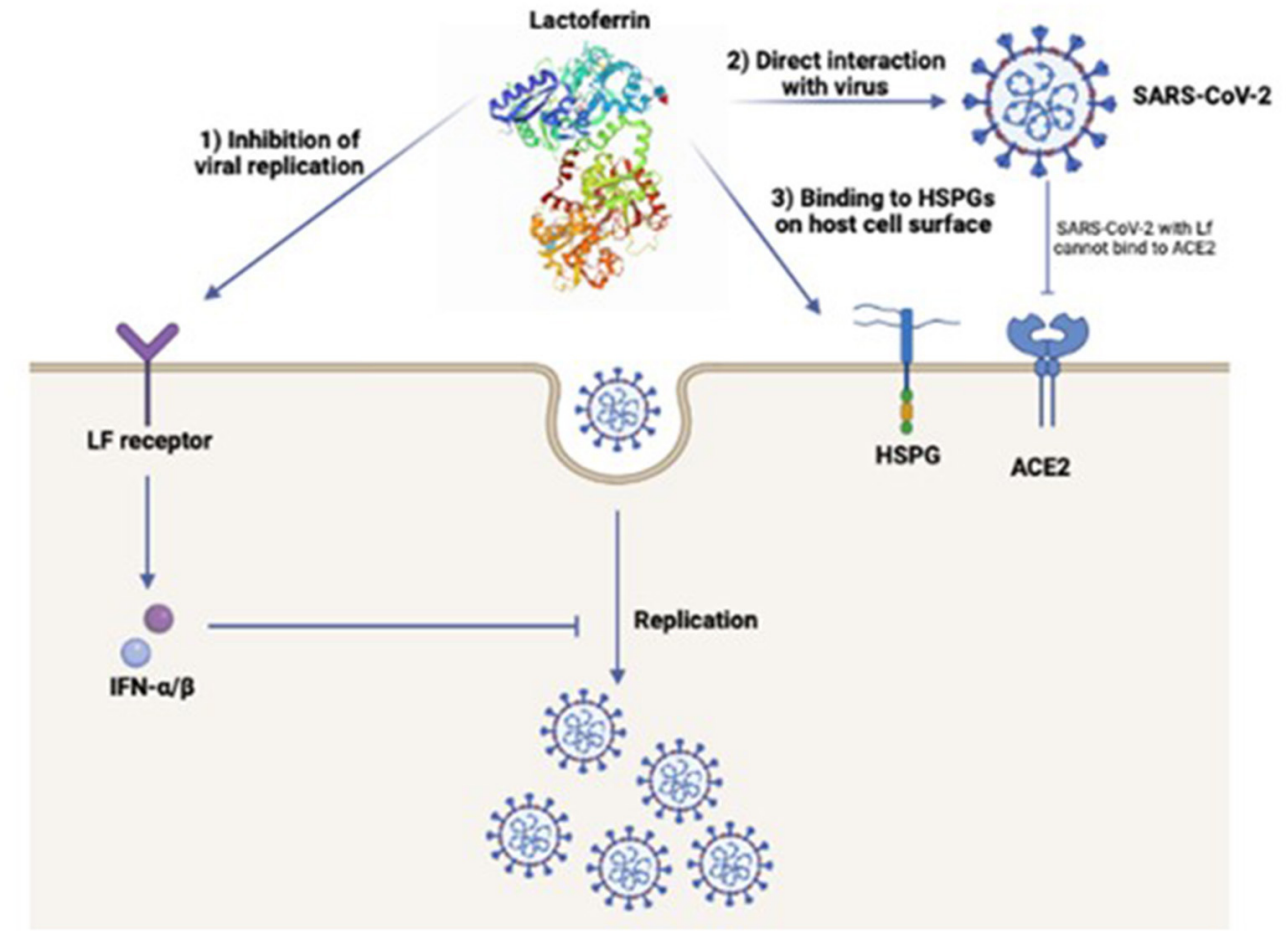
Potential mechanisms of lactoferrin antiviral mechanism against SARS-CoV-2; (1) Inhibition of viral replication *via* the induced α and β IFN by direct binding of lactoferrin to its cell receptor, (2) Direct interaction of lactoferrin with SARS-CoV-2 prevents the binding of the virus to ACE2 receptor, (3) Binding of lactoferrin to HSPGs on the host cell surface which prevents the viral entry through the host cell (43, 99).

Several studies have been performed to understand the potential antiviral mechanisms of Lf against SARS-CoV-2 (Figure 1) (43, 99). A recent study suggested potential antiviral mechanisms of Lf against viruses including SARS-CoV-2 (99). One of these includes heparan sulfate proteoglycans (HSPGs) which increase the virus aggregation at the cell surface and enhance their specific receptor binding abilities. Several similar studies also suggested that Lf binds to HSPGs and prevents viral entry to the host cell (80, 99, 100). The other possible mechanism that indicates, Lf can link to SARS-CoV-2 directly thus, Lf prevents the binding of the virus to its receptor ACE2. Furthermore, Lf can also induce α and β interferon (IFN) *via* intracellular cell signals, *via* Lf receptors which inhibit the viral replication after the virus entry to the cell. This mechanism is considered a significant factor in the early stages of viral infection (82).

Several *in-vitro* and *in-vivo* studies related to the impact of Lf on a broad range of viruses from HIV to SARS-CoV-2 were conducted as shown in Table 2 (99). These studies have shown Lf can inhibit various viruses by using distinct mechanisms such as inhibition of viral entry, cytopathic effect, binding, replication, reduction of the severity, and antiviral effect.

Regarding Lf's antiviral effect against SARS-CoV-2, some significant studies have been conducted based on the analysis of different parameters (73, 81, 101, 102). In one of these *in-vivo* studies, 75 patients with a positive result at IgM/IgG rapid test have participated. Each patient took liposomal bLf syrup (32 mg of Lf/10 ml) with four to six doses, each dose containing 10 mg/10 ml syrup, every day for 10 days. The patients who were given syrup were observed for 10 days at least twice a day then they were investigated again after 30 days. As a result of the investigation, Lf treatment had shown a positive effect and all patients had a faster recovery when compared with the control group (81).

In another *in-vitro* study examining the Lf's protective effect against SARS-CoV-2, the gene responsible for the antiviral immune response was detected by qRT-PCR in uninfected Caco-2 intestinal cells with Lf. Both Lf treated and non-treated Caco-2 cells were infected by SARS-CoV-2. With the results of qRT-PCR, expression of some specific genes was observed, which are pattern recognition receptors gene—toll-like receptor (TLR3 and TLR7), inform regulatory factor gene (IRF3 and IRF7) with helicase C domain 1 and mitochondrial anti-viral signaling factor (MAVS). All genes are critical for sensing RNA viruses, and results indicated that Lf partially managed to inhibit SARS-CoV-2 infection (101).

In another study considering the saliva analysis of COVID-19 patients (102), three groups of participants were selected, which were either non-infected or infected before 7 days or recovered from the virus for at least 2 months. All saliva samples of participants were analyzed by real-time PCR for the detection of SARS-CoV-2. In addition, several parameters including TNF-α, IL-6, IL-10 (Cytokines), Lf, lysozyme, IgG, IgA, and IgM were analyzed by the ELISA method. In the results, the Lf amount was recorded lower in the infected patients when compared with the non-infected group. This indicates that there might be a relationship between the observed cytokine storm—an aggressive inflammatory response—and a decrease of Lf in the COVID-19 patients. The indication is also supported in another review article about Lf's potential reducing effect on SARS-CoV-2 induced cytokine storm (15). Lf's immune system modulating function might control the excessive stimulation of immune system that is led to creation of cytokine storm.

A study, oral and intranasal liposomal bLf were tested in 92 patients with mild, moderate severity, or asymptomatic COVID-19 (73). The patients were divided into three groups in total with 32 patients in the first, 28 patients in the second, and 32 patients in the third group. The patients in the first group were given the capsules and nasal sprays three times per day. In the first group, 14 out of 32 patients were treated at the hospital. The remaining 18 patients were treated with oral capsules (100 mg) and intranasal (8 mg/ml) bLf at home. The 28 patients in the second group were kept under surveillance at home without any anti-COVID-19 drug. During the monitoring, the condition of four patients worsened and they were hospitalized. Finally, the third group with 32 patients was treated in the hospital with hydroxychloroquine and lopinavir (SOC). The third group of patients took two capsules (200/50 mg) of lopinavir twice and hydroxychloroquine (200 mg) capsules per day, respectively. According to the results, in the group treated with liposomal bLf, the COVID-19 PCR test of individuals was negative after an average of 14.25 ± 6.0 days. In addition, the group of patients treated with SOC was negative for the COVID-19 PCR test after a mean of 27.13 ± 14.4 days. On the other hand, patients who were not treated in any way were negative for COVID-19 PCR tests at the end of 32.61 ± 12.2 days. Furthermore, symptoms such as coughing, headache, inability to smell (anosmia), and myalgia had developed in patients treated with SOC at the end of the study, whereas patients treated with bLf had not. Normally, COVID-19 patients have a high level of IL-6. When bLf was used to treat COVID-19 patients, IL-6 levels had significantly reduced until the end of the study, and similarly, the same reductions had been observed in D-dimer serum and ferritin levels.

## Conclusion

Apart from its intrinsic antiviral activity and immune system boosting property, Lf has a great effect on the iron levels of specific tissues and thus affects many iron-containing and oxygen-dependent factors in the organism. Lf's multifunctional property might be effectively implemented into the treatment procedure for COVID-19. Its antiviral effects and influence on iron homeostasis might be integrated into the infection of SARS-CoV-2, just like in other viruses. Thus, Lf-based treatment strategies have great untapped potential in many viral infections including COVID-19.

Due to Lf's critical and clear influence on many infections, the mechanisms behind this antiviral characteristic must be investigated in detail. The potential mechanisms that Lf might possess can point to evidence and explanation of Lf's prevention effect on SARS-CoV-2. In addition, Lf not only carries potential treatment agents against SARS-CoV-2 infection, but it also has a specialty in recovery with its influence on the overall immune system. In the future, Lf not only can be used as a therapeutic agent, but also can be used as a recovery supplement for patients infected with COVID-19.

Despite the sudden peak in the related studies in the literature in the last 3 years due to the pandemic, Lf still hasn't gotten the attention it deserves in the COVID-19 studies. Although the main focus of COVID-19 studies is on the prevention with vaccines, the effect of Lf on the immune system to prevent COVID-19 and the synergistic studies with vaccines are almost non-existent. The previous studies on Lf mainly focus on the treatment potential of Lf. On the other hand, despite the early studies on Lf effects on COVID-19 studies not only put forth promising results but also showed comparable or even better results than traditional treatment strategies such as SOC, there are still not enough studies on the treatment potential of this multifunctional protein.

Due to the many successful studies and adopted strategies with other similar and dissimilar viral infections, current literature not only strongly suggests the great potential of Lf in COVID-19 treatment but also implies the great potential of the molecule in the prevention of the disease.

## Author contributions

SK organized the general content of the paper. EB and FE were responsible for general editing and organizing the authors as well as the contribution for two sections. MK, HD, and AA contributed one section of the paper. SS and AŞ were responsible for editing and organizing the paper. All authors contributed to the article and approved the submitted version.

## Funding

Uluova Süt Ticaret A.S. (Uluova Milk Trading Co.) and TUBITAK #118z146 have funded this study. The funder was not involved in the study design, collection, analysis, interpretation of data, the writing of this article or the decision to submit it for publication.

## Conflict of interest

Author SK has received funding from Uluova Süt Ticaret A.S. (Uluova Milk Trading Co.). The funder was not involved in the study design, collection, analysis, interpretation of data, the writing of this article or the decision to submit it for publication. The remaining authors declare that the research was conducted in the absence of any commercial or financial relationships that could be construed as a potential conflict of interest.

## Publisher's note

All claims expressed in this article are solely those of the authors and do not necessarily represent those of their affiliated organizations, or those of the publisher, the editors and the reviewers. Any product that may be evaluated in this article, or claim that may be made by its manufacturer, is not guaranteed or endorsed by the publisher.
